# Retinoic acid promotes *in vitro* follicle activation in the cat ovary by regulating expression of matrix metalloproteinase 9

**DOI:** 10.1371/journal.pone.0202759

**Published:** 2018-08-24

**Authors:** Mayako Fujihara, Kohei Yamamizu, Pierre Comizzoli, David E. Wildt, Nucharin Songsasen

**Affiliations:** 1 Center for Species Survival, Smithsonian Conservation Biology Institute, National Zoological Park, Front Royal, Virginia, United States of America; 2 Wildlife Research Center, Kyoto University, Kyoto, Japan; 3 Laboratory of Genetics, National Institute on Aging, National Institutes of Health, Baltimore, Maryland, United States of America; Faculty of Animal Sciences and Food Engineering, University of São Paulo, BRAZIL

## Abstract

Retinoic acid (RA) facilitates tissue morphogenesis by regulating matrix matalloproteinase (MMPs) expression. Our objective was to examine the influence of RA on *in vitro* development of follicles enclosed within domestic cat ovarian tissues. Ovarian cortices from 9 prepubertal and 13 adult cats were incubated for 7 d in medium containing 0 (control), 1 or 5 μM RA and then analyzed for viability. Cortices from additional three animals of each age group were cultured in the same condition and follicle morphology, stage and size were histologically evaluated. In a separate study, cortices from 14 donors (7 prepubertal; 7 adult cats) were incubated in 0 or 5 μM RA for 7 d and assessed for (1) *MMP1*, *2*, *3*, *7*, *9* and *TIMP1* expression by qPCR and (2) protein expression of MMP9 by immunohistochemistry. Donor age did not influence follicle response to RA. Collective data from both age groups revealed that percentages of primordial follicles in 5 μM RA treatment were lower (*P* < 0.05; 40.5 ± 4.5%) than in fresh cortices (66.7 ± 5.3%) or controls (60.1 ± 4.0%) with 1 μM-RA treatment producing intermediate (56.3 ± 4.0%) results. Proportion of primary follicles in 5 μM RA (21.7 ± 3.3%) was higher than in fresh cortices (4.9 ± 2.9%) and controls (9.0 ± 2.8%) with 1 μM-RA treatment producing an intermediate value (13.8 ± 2.0%). Furthermore, proportion of secondary follicles increased after 7 d in the presence of 5 μM RA (9.5 ± 2.7%) compared to other groups (fresh, 1.9 ± 0.8%; control, 2.6 ± 1.1%; 1 μM RA, 2.5 ± 0.2%). *MMP9* transcript and protein were upregulated, whereas *MMP7* mRNA was suppressed by 5 μM-RA treatment compared to fresh counterparts. RA did not impact *MMP1*, *2*, *3*, *13* or *TIMP1* expression. In summary, RA activated cat primordial follicle growth likely via a mechanism related to upregulation of *MMP9* and down-regulation of *MMP7* transcripts.

## Introduction

Primordial follicle activation and growth progression beyond the primary follicle stage require locally-produced factors and peptides independent of pituitary gonadotropins [1] as well as support from surrounding somatic cells. Under these paracrine influences, a follicle undergoes a marked increase in surface area as it transitions from the primordial stage to the primary, secondary and, ultimately, preovulatory Graafian stage. During this development, there is continual remodeling of the follicle’s wall and the adjacent extracellular matrix (ECM) resulting in collagen turnover in the theca externa and the surrounding ovarian stroma, all permitting follicular expansion [2,3]. The matrix metalloproteinases (MMPs), a family of zinc-containing enzymes, appear to have a significant responsibility for these changes [2,3]. mRNA and protein expression MMPs and TIMP, its tissue inhibitor, have been demonstrated in the ovary of multiple mammalian species, including the mouse, cow, pig, sheep and human [3]. We recently reported expression of *MMP1*, *MMP2*, *MMP3*, *MMP7*, *MMP9*, *MMP13* and *TIMP1* mRNA in the ovary of the domestic cat with abundance and expression pattern of these enzymes varying during folliculogenesis [4]. Specifically, *MMP1*, *2*, *3* and *9* mRNA increase multiple fold from a primordial nadir to peak in follicles forming an antral cavity. Meanwhile, *MMP7* transcripts increase 2-fold between the primordial and primary stage and then plateau, whereas *MMP13* mRNA peaks in the primary follicle (2.5-fold above baseline), but then is lower in more advanced counterparts. Lastly, *TIMP1* sharply increases (6-fold) in the secondary follicle stage, but gradually declines thereafter. Collectively, these findings reveal that there is dynamic and rather striking variation in various MMP enzymes over the course of the folliculogenic process, suggesting different roles during follicular maturation.

Retinoic acid (RA), a vitamin A metabolite, has been shown to be involved in tissue morphogenesis, cellular proliferation, differentiation and apoptosis in multiple cell/tissue types during embryogenesis and organogenesis [5,6]. This compound also is known to influence MMP expression in many cell types. For example, a study of human neuroblastoma cells has demonstrated that RA induces neuronal cellular differentiation by up-regulating MMP9 expression [6]. By contrast, it has been shown that RA down-regulates protein expression of MMP2 and 9 that, in turn inhibits proliferation and migration of human arterial smooth muscle *in vitro* [7]. There also is evidence of RA influence on reproductive cells; examples include, promoting oocyte maturation in the cow [8] and embryo quality in the human [9]. However, the impact of RA on ovarian follicle development has not been investigated.

We study the domestic cat as a research model for two reasons. First, as a physically larger species than conventional laboratory rodents, the cat offers more, analogous reproductive complexities to women, including similarities in follicle and oocyte size and nuclear configuration [10]. Secondly, what has been learned about the domestic cat has application to comparative studies, and sometimes improved conservation, of a variety wild Felidae species, many of which are vulnerable to extinction [11]. In both cases, we have been keen to thoroughly understand what regulates ovarian function, especially follicle development *in vitro* as a mean of rescuing the maternal genome represented in thousands of follicles that never fully develop and, thus, never produce a viable, fertilizable oocyte [4,10,12,13]. Given this long-term aim as well as knowledge that RA has cell promoting abilities [5–9], our objective was to examine, for the first time, the influences of RA on *in vitro* folliculogenesis, specifically through an impact on MMPs manifestation in ovarian tissue. Therefore, our hypothesis was that supplementing *in vitro* culture environment with RA stimulates ovarian follicle development by altering MMP expression. Because earlier work in our laboratory demonstrated a donor age effect on ability of ovarian stromal cells to proliferate *in vitro* [13], we also compared the influence of RA on follicles from prepubertal versus adult females.

## Materials and methods

### Chemicals

All chemicals were purchased from Sigma-Aldrich (St Louis, MO) unless otherwise indicated.

### Collection and *in vitro* culture of ovarian cortices

The source of ovaries was known age prepubertal (5–6 mo old, n = 19) and adult (8 mo-3 yr old, n = 23) domestic cats (domestic short hair) in good health condition that had undergone routine ovariohysterectomy at local veterinary clinics throughout the year. Upon excision of the reproductive tract, each ovarian pair was removed from the oviduct/uterine cornus, immersed in L-15 medium (containing 10 mM HEPES, 100 μg/ml penicillin G sodium and 100 μg/ml streptomycin sulfate) and transported in a 4°C container to the laboratory within 1 to 6 hr. Ovaries of adult cats were obtained during inter-estrous period based on the absence of corpora lutea and preovulatory follicles on both ovaries.

Ovarian cortical slices (1 mm thick) were dissected from the surface of each ovary using a surgical blade and then sectioned in equal pieces (1–1.5 mm width) [12,13]. To avoid the influence of different intra-ovarian condition, tissue from both ovaries was combined for each donor and then incubated (38.5°C, 5% CO_2_, humidified air for 7 d) separately from other donors. The basic culture medium used was standard for the domestic cat as developed for earlier studies [12,13]. The base medium was Eagle’s MEM supplemented with 0.4 μg/ml insulin, 0.4 μg/ml transferrin, 0.5 ng/ml selenium, 2 mM L-glutamine, 100 μg/ml penicillin G sodium, 100 μg/ml streptomycin sulfate, 0.05 mM ascorbic acid, 10 ng/ml porcine FSH (Folltropin-V, Bioniche Animal Health, Belleville, ON, Canada), 0.1% (w/v) polyvinyl alcohol and 100 ng/ml EGF [13]. To ensure freshness and eliminate metabolic waste, half the volume of the culture medium was exchanged every 48 hr throughout the 7 d study interval.

‘The Smithsonian Conservation Biology Institute’ Animal Care and Use Committee has granted a waiver of the animal care and use approval because the tissues were spayed materials that otherwise would be discarded.

### Assessment of follicle viability

Follicle viability within the ovarian cortical tissues was evaluated at Day 0 (fresh control; day of tissue excision and initial incubation) or Day 7 of *in vitro* culture using calcein AM/ethidium homodimer-1 staining (Invitrogen) and observation under a fluorescent microscope (Olympus BX40; Olympus America Inc., Central Valley, PA). Follicles were considered viable when both the oocyte and surrounding granulosa cells fluoresced green by calcein. For each replicate, at least two cortical pieces were assessed for each fresh and culture treatments, with all follicles in a given piece counted regardless of size.

### Histological analysis and classification of follicular structure

Histological analysis and classification of follicle structure post-treatment (see Experimental Design below) were performed as described previously with minor modifications [10,11]. Briefly, pieces of fresh and cultured ovarian tissues were fixed in Bouin’s solution, maintained at 4°C overnight and fixative were changed to 70% ethanol and kept at 4°C until being processed for histological analysis. Briefly, the fixed tissues were dehydrated in a graded series (70%-100%) of ethanol solutions and then embedded in paraffin. Serial sections (5 μm thickness) of each cortical piece were cut and stained with hematoxylin (American MasterTech, Lodi, CA) and eosin (American MasterTech). Tissue recovered from the same donor cat were processed on the same day. Only follicles containing oocytes with a visible nucleus were assessed. For each ovarian piece, three sections (including the largest area at the center and one piece before and another after, each at least 20 μm apart) were assessed by light microscopy (Olympus BX40) to determine the percentage of structurally normal follicles, at each follicular stage (follicle distribution), as well as their density and size. More specifically, all follicles within a cortical piece were characterized as ‘normal’ (when the nucleus of the oocyte and the surrounding granulosa cells were structurally intact) or ‘abnormal’ (oocyte and/or granulosa cells contained a pyknotic, fragmented or shrunken nucleus) [12]. Percentages of structurally-normal follicles per section were calculated by dividing the number of normal follicles by total number follicles evaluated. Follicle density was defined as total numbers of morphologically-normal follicles in 1 mm^2^ of ovarian cortex as assessed by IPLab imaging software (BDBioSciences, San Jose, CA). Each follicle was further classified as (1) primordial (one layer of flattened granulosa cells around the oocyte), (2) transitioning from primordial to primary (a single mixed layer of flattened and cuboidal granulosa cells around the oocyte), (3) primary (a single layer of exclusively cuboidal granulosa cells around the oocyte) or (4) secondary (two or more layers of cuboidal granulosa cells) using criteria previously published by our laboratory [12]. Follicular diameter was defined as the maximum diameter measured within the basal membrane.

### RNA isolation and quantitative reverse transcription polymerase chain reaction (qPCR)

Expression of *MMP1*, *2*, *3*, *7*, *9* and *13*, as well as *TIMP1*, was determined by qPCR using fresh and cultured ovarian tissue, as described previously [4]. Five to six freshly-collected ovarian cortices (Day 0) from each animal as well as cortical pieces incubated for 7 days in the Control (0 μM RA) or 5 μM RA treatment were lysed in Trizol (Invitrogen, Carlsbad, CA) and stored at -80°C until RNA extraction. Reverse-transcription was performed with a SuperScript III first-strand synthesis system (Invitrogen) according to manufacturer’s instructions. For each animal, qPCR was completed in triplicate using Power SYBR Green PCR Master Mix (Life Technologies, Carlsbad, CA) and a 7300 real time PCR system (Life Technologies). PCR amplification was conducted with 40 cycles (95°C for 15 s and 60°C for 60 s). Amplification and melting curves of each PCR product were checked to verify efficiencies and the targeted amplicon. The CT value of each gene was normalized against the average CT of glyceraldehyde-3-phosphate dehydrogenase (*GAPDH*, housekeeping gene) to generate a delta CT (ΔCT) [14]. Target gene expression levels were determined by the comparative threshold cycle (ΔCt) method [14]. Statistical analyses between ages and among treatment groups were performed based on 2^-ΔCT^. Data were expressed as the relative mRNA expression given by the mean (±standard error of mean [SEM] of 2^-ΔCT^ and fold change over fresh tissues given by the mean (±SEM) of 2^-ΔΔCT^. Primers for specific transcripts were designed using Primer3 software with all sequences listed in Table 1.

**Table 1:** Primers for quantitative reverse transcription PCR (qPCR).

Primers		Sequence
Cat MMP1	Forward	ttcggggagaagtgatgttc
	Reverse	caagtccatttggcaggttt
Cat MMP2	Forward	cttgaccagagcacgattga
	Reverse	agatcaggcgtgtagccaat
Cat MMP3	Forward	tgactcgaaggttgatgctg
	Reverse	tgtcactttctttgcgttgg
Cat MMP7	Forward	acttgccatccagaaacagg
	Reverse	agtgggatctctttgctcca
Cat MMP9	Forward	gcagctggcagaggaatatc
	Reverse	cagggtggttctgtccagtt
Cat MMP13	Forward	gactttccagggattggtga
	Reverse	aatacggttgctccagatgc
Cat TIMP1	Forward	gcgaagaatgcaccgtattt
	Reverse	cttgtcagtgcctgtgagga
Cat GAPDH	Forward	ctcatgaccacagtccatgc
	Reverse	gtgagcttcccattcagctc

### Localization of MMP9 protein in fresh and cultured ovarian tissues

Protein expression of MMP9 was assessed using the immunohistochemistry procedure that relied on the ImmunoCruz rabbit LSAB staining system (Santa Cruz Biotechnology, Santa Cruz, CA), as described previously [4]. Briefly, three to five fresh and cultured ovarian cortical pieces from each donor were immersed in Bouin’s fixative, embedded in paraffin, sectioned (5 μm thickness), dewaxed, rehydrated and then boiled for 20 min in a buffer (pH 6.2) containing 10 mM citric acid, 2 mM EDTA and 0.1% Tween. Each section then was incubated with 3% (v/v) H_2_O_2_ in PBS for 10 min, blocked with 1.5% goat serum and 1% BSA in PBS for 30 min and then exposed (4°C, overnight in a moist chamber) to rabbit anti-MMP9 as the primary antibody (Thermo Fisher Scientific, Waltham, MA) in 0.3% Triton-X (diluted 1:100). After washing with PBS, each section was incubated with anti-rabbit immunoglobulin G antibody conjugated with Biotin (Santa Cruz Biotechnology) as a secondary antibody for 1 hr at room temperature (~22°C) followed by HRP-streptavidin complex (Santa Cruz Biotechnology) for 1 hr (~22°C). Finally, each section was stained with HRP substrate and counterstained with hematoxylin (both from Santa Cruz Biotechnology) followed by evaluation under an Olympus BX 40 microscope for protein distribution. Control (negative) sections were incubated with normal rabbit IgG antibody (Santa Cruz Biotechnology) in PBS rather than the primary antibody. Degree of staining in all sections was graded subjectively as very strong (+++), strong (++), positive (+) or weak/varied (±).

### Experimental design

#### Study 1: Influence of RA on follicle growth—Study 1a: Influence of RA on follicle viability

To determine the effect of RA on *in vitro* follicle development, ovarian cortical pieces from prepubertal (n = 9) versus adult (n = 13) cats were incubated separately for 7 d on 1.5% (w/v) agarose gel blocks in protein-free medium within a 24 well culture plate (Corning Incorporated, Corning, NY), as described previously [12]. The ovarian cortical pieces combined from both ovaries were randomly divided into fresh, non-cultured control or three culture treatment groups. The culture medium (as described above) was supplemented with 0 (control), 1 or 5 μM RA (2–5 cortical pieces/cat/treatment). Cortical pieces of each donor cat were assessed for viability on the same day of sample collection (Day 0) or after 7 d of culture.

#### Study 1: Influence of RA on follicle growth—Study 1b: Influence of RA on follicle structure and distribution

Tissues from different three cats of each age group were cultured for 7d with 0 (control), 1 or 5 μM RA and assessed for follicle morphology, stage and size as described above. Fresh tissues from each donor cat were fixed and assessed for the same parameters as served as fresh control. Fixed tissue obtained from the same donor were processed for histological evaluation on the same day.

#### Study 2: Influence of RA on MMPs and TIMP mRNA expression

Based on findings from Study 1, we investigated the influence of RA on *MMP1*, *2*, *3*, *7*, *9* and *13* as well as *TIMP1* expressions. Ovarian cortical pieces from four prepubertal and four adult cats were divided into fresh control or two culture groups for each donor. For cultured treatments, cortices were incubated with either 0 (control) or 5 μM RA (5–6 pieces/cat/treatment) for 7 d. Ovarian cortical pieces were lysed and stored in Trizol immediately after the collection in fresh (same donors of culture tissues) or after 7 d of culture. The cultured tissues as well as the fresh control from same donors were then processed together for real-time qPCR (as described above).

#### Study 3: Influence of RA on MMP9 protein expression

Based on Study 2 results, we then explored the influence of RA on MMP9 protein expression. Ovarian tissues recovered from three additional prepubertal and three adult cats were randomly divided into fresh controls or two culture treatment groups for each donor and incubated with 0 (control) or 5 μM RA (as described above). Tissue from these cultures plus fresh controls (same donors of culture tissues) were fixed on the same day of collection and examined together for MMP9 protein expression by immunohistochemistry (as described above).

### Statistical analysis

Averaged data are presented as means ± SEM. A Shapiro-Wilk test was used to evaluate normality of the dataset and a Bartlett test to confirm homogeneity of variances. The proportions of each follicle stage between the two age groups were assessed by an unpaired, one-tailed Student’s t-test. Differences in viability, morphology among cultured treatments were evaluated by analysis of variance (ANOVA) followed by a Newman-Keuls Multiple Comparison test. The differences of mRNA expression (2^-ΔCT^) within a given treatment between the two age groups were assessed using an unpaired, one-tailed Student’s t-test. Contrasts in mRNA expression among fresh and cultured treatments were performed using ANOVA followed by a Turkey’s Multiple Comparison test. Differences were considered significant at *P* < 0.05 (GraphPad Prism ver. 4.00, GraphPad Software, La Jolla, CA).

## Results

### Study 1: Influence of RA on follicle growth

There were no differences (*P* > 0.05) in follicular distribution in fresh cortices recovered from prepubertal versus adult donors as well as among age groups. In cortices from prepubertal cats, 70.7 ± 9.1%, 26.5 ± 6.4% and 2.3 ± 2.3% were primordial, in transition from primordial to primary and primary stage follicles, respectively. Respective values from adult counterparts were 62.9 ± 4.6%, 33.7 ± 3.7% and 3.4 ± 1.1%. There also was no influence (*P* > 0.05) of donor age on the morphological normality (i.e., there were comparable mean percentages of morphologically-normal follicles per section.) of incubated follicles in all culture treatments. Therefore, data from the two age groups were combined for viability and histological assessments of the impact of RA on cultured ovarian tissue.

#### Study 1a: Influence of RA on follicle viability

**Fig 1:**
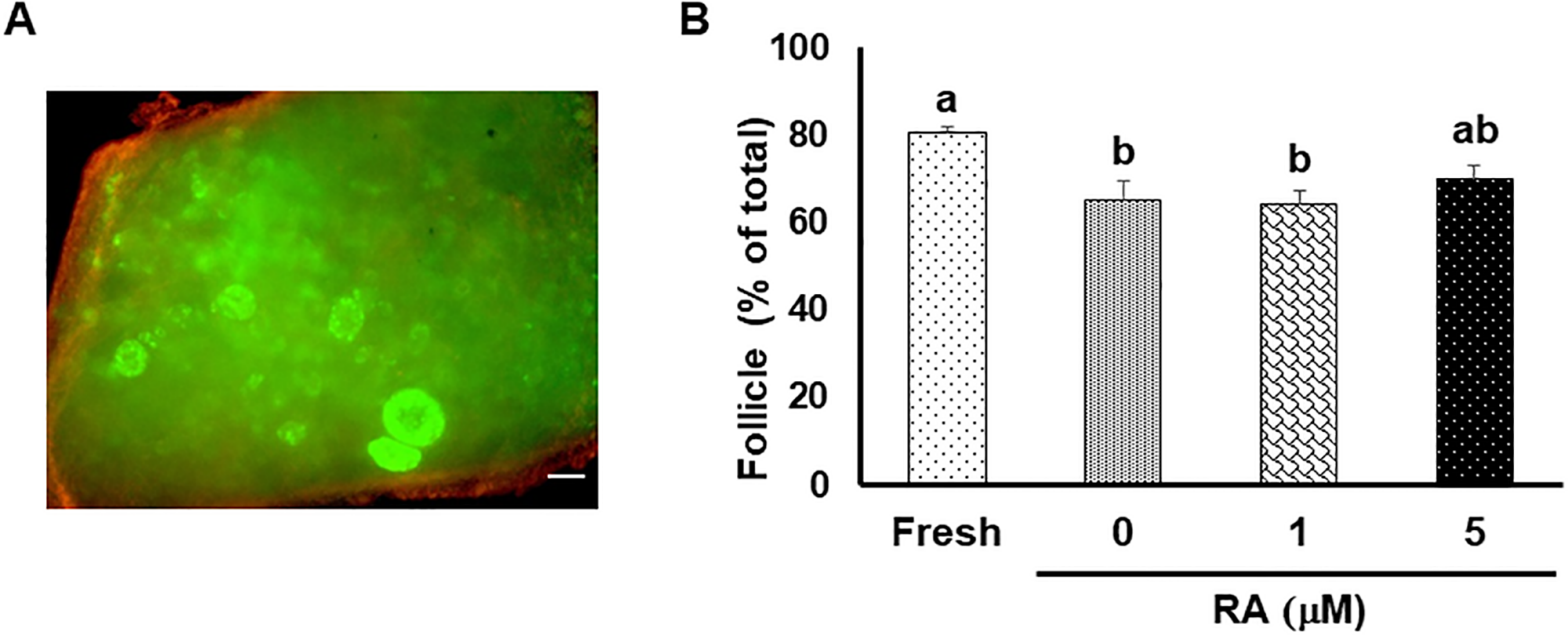
Influence of RA on follicle viability. (A) Fluorescent staining with calcein-AM/ethidium homodimer-1 after culture in 5 μM retinoic acid for 7 d. Green fluorescence indicates viable follicles/cell. Bar = 100 μm. (B) Mean (± SEM) of follicle viability in freshly collected cat ovarian tissue and cortical pieces cultured for 7 d. Different letters indicate significant (P < 0.05) differences.

Based on calcein-AM/ethidium homodimer staining, most of the follicles (> 80%, Fig 1) were viable at the onset of collection. Follicular viability decreased (*P* < 0.05) after *in vitro* culture with 0 or 1 μM RA treatment, but was similar in cortices incubated with 5 μM RA (Fig 1).

#### Study 1b: Influence of RA on follicle morphology and distribution

**Fig 2:**
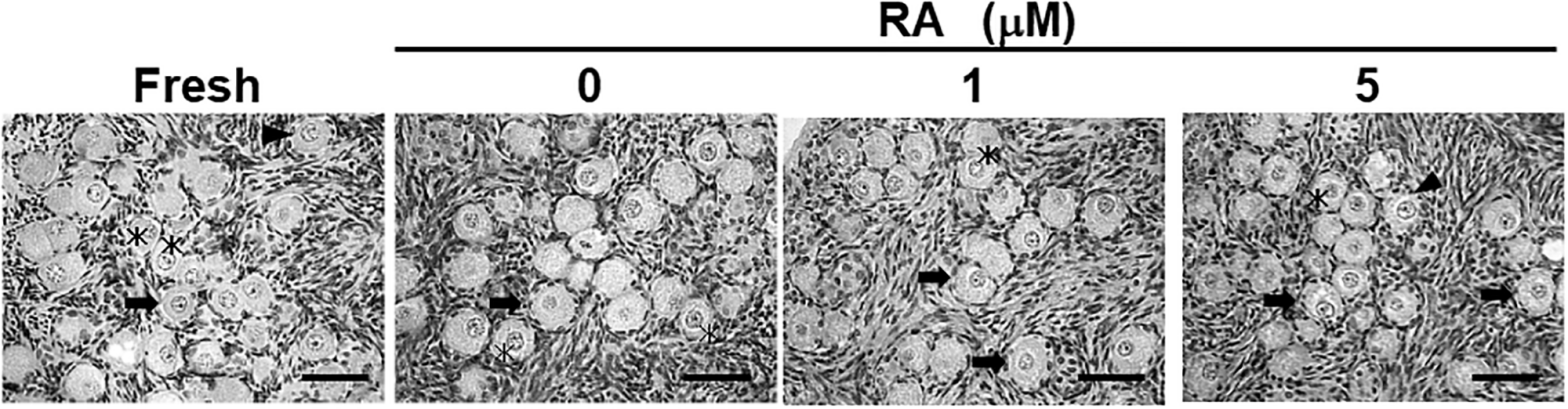
Histomicrographs of ovarian tissue cultured for 0 (fresh) and 7 d with RA. Bar = 50 μm. Asterisks indicate primordial follicles; arrows indicate the transition stage of primordial to primary follicle; arrow heads indicate primary follicles.

Histological analysis revealed that proportions of morphologically-normal follicles were comparable (*P* > 0.05) between fresh and incubated tissue as well as among culture treatments (Fig 2, Table 2). Furthermore, follicle density within each cortical piece was similar (*P* > 0.05) between fresh (35.4 ± 13.3 follicles/mm^2^) and cultured tissues (control, 37.7 ± 8.7; 1 μM RA, 31.5 ± 3.9; 5 μM RA, 23.2 ± 3.3 follicles/mm^2^) (Table 2). However, the presence and concentration of RA influenced (*P* < 0.05) follicle distribution in a dose-dependent fashion by 7 d of culture. Specifically, the proportion of primordial follicles in cortices incubated with 5 μM RA (40.5 ± 4.5%) was less (*P* < 0.01) than that of fresh tissue (66.7 ± 5.3%) and the control (60.1 ± 4.0%) with the values for 1 μM being intermediate (56.3 ± 4.0%) (Table 3). The percentage of follicles in transition from the primordial to primary stage did not differ (*P* > 0.05) among treatments (Table 3). However, the proportion of primary follicles increased (*P* < 0.01) in 5 μM RA (21.7 ± 3.3%) compared to fresh cortices (4.9 ± 2.9%) and the control (9.0 ± 2.8%) with the 1 μM RA treatment produced an intermediate value (13.8 ± 2.0%) (Table 3). Furthermore, the proportion of secondary follicles increased (*P* < 0.01) by 7 d in the presence of 5 μM RA (9.5 ± 2.7%) compared to 1 μM RA (2.5 ± 0.2%), fresh cortices (secondary, 1.9 ± 0.8%) and the control (secondary, 2.6 ± 1.1%) (Table 3).

**Table 2:** Influence of RA on follicle densities and morphology.

	Fresh	0 μM RA	1 μM RA	5 μM RA
**Follicle densities (follicle/mm**^**2**^**)**	**35.4** ± **13.3**	**37.7** ± **8.7**	**31.5** ± **3.9**	**23.0** ± **3.3**
**Normal Follicles (%)**	**64.3** ± **7.5**	**53.9** ± **4.3**	**58.2** ± **5.6**	**52.3** ± **5.5**

Mean (± SEM) percentages of morphologically-normal follicles for ovarian tissue cultured for 0 (fresh) and 7 d with 0 (control), 1 or 5 μM RA.

**Table 3:** Influence of RA on follicle distribution.

Follicular stage	Fresh	0 μM RA	1 μM RA	5 μM RA
**Primordial**	**66.8** ± **4.9**^**a**^	**60.1** ± **5.1**^**a**^	**56.3** ± **3.8**^**ab**^	**40.5** ± **5.2**^**a**^
**Transition**	**26.4** ± **3.7**	**28.4** ± **2.5**	**27.4** ± **2.9**	**31.2** ± **4.0**
**Primary**	**4.9** ± **2.9**^**a**^	**9.0** ± **2.8**^**a**^	**13.8** ± **2.0**^**ab**^	**21.7** ± **4.3**^**b**^
**Secondary**	**1.9** ± **0.8**^**a**^	**2.6** ± **1.1**^**a**^	**2.5** ± **0.2**^**a**^	**9.5** ± **2.6**^**b**^

Mean (± SEM) percentages of follicle distribution across each stage (primordial, transition from primordial to primary, primary and secondary) for ovarian tissue cultured for 0 (fresh) and 7 d with 0 (control), 1 or 5 μM RA. Within rows, different letters indicate differences (*P* < 0.05) in the percentages of each follicular stage.

Follicle diameter tended to be larger after culture, but the influence of RA treatment varied depending on developmental stages. Follicle diameter of primordial stage was greater (*P* < 0.05) in cortices incubated with 0 (control, 63.9 ± 1.5 μm) or 1 μM RA (64.9 ± 1.1 μm) compared to fresh tissues (58.4 ± 1.2 μm), whereas the tissue incubated with 5 μM RA group contained intermediate (*P* > 0.05) follicle size (61.6 ± 1.4 μm) (Table 4). The diameter of transition stage increased in all cultured tissues regardless of culture treatment (control, 69.2 ± 1.2; 1 μM RA, 69.2 ± 1.0; 5 μM RA, 68.1 ± 1.8 μm) compared to fresh tissues (fresh, 62.3 ± 2.4 μm) (Table 4). The diameter (80.9 ± 1.2 μm) of primary follicles incubated with 1 μM RA was greater (*P* < 0.05) than that of fresh (67.5 ± 6.1 μm) and other incubated cortices (control, 74.7 ± 1.8; 5 μM, 70.9 ± 2.9 μm) (Table 4).

**Table 4:** Influence of RA on follicle diameter.

Follicular stage	Fresh	0 μM RA	1 μM RA	5 μM RA
**Primordial**	**58.4** ± **1.2**^**a**^	**63.9** ± **1.5**^**b**^	**64.9** ± **1.1**^**b**^	**61.6** ± **1.4**^**ab**^
**Transition**	**62.3** ± **2.4**^**a**^	**69.2** ± **1.2**^**b**^	**69.2** ± **1.0**^**b**^	**68.1** ± **1.8**^**ab**^
**Primary**	**67.5** ± **6.1**^**a**^	**74.7** ± **1.8**^**ab**^	**80.9** ± **1.2**^**b**^	**70.9** ± **2.9**^**a**^
**Secondary**	**105** ± **8.4**	**104.2** ± **11.4**	**126.4** ± **16.4**	**100.3** ± **9.0**

Mean (± SEM) of follicle diameters (μm) in each stage for fresh ovarian cortices and tissue cultured for 7 d in the presence of 0, 1 or 5 μM RA. Within rows, different letters indicate differences (*P* < 0.05) in the each follicular stage.

### Study 2: Influence of RA on MMPs and TIMP mRNA expression

Age did not influence mRNA expression of all genes, except for *MMP2*. Specifically, *MMP2* expression of prepubertal tissues incubated in 5 μM RA was lower (P < 0.05) than in adult cortices cultured under the same condition. Because there were no differences in *MMP7*, *MMP9*, *MMP13* and *TIMP1* transcripts between the two age groups, data were combined and analyzed to determine the impact of RA treatment on gene expression.

**Fig 3:**
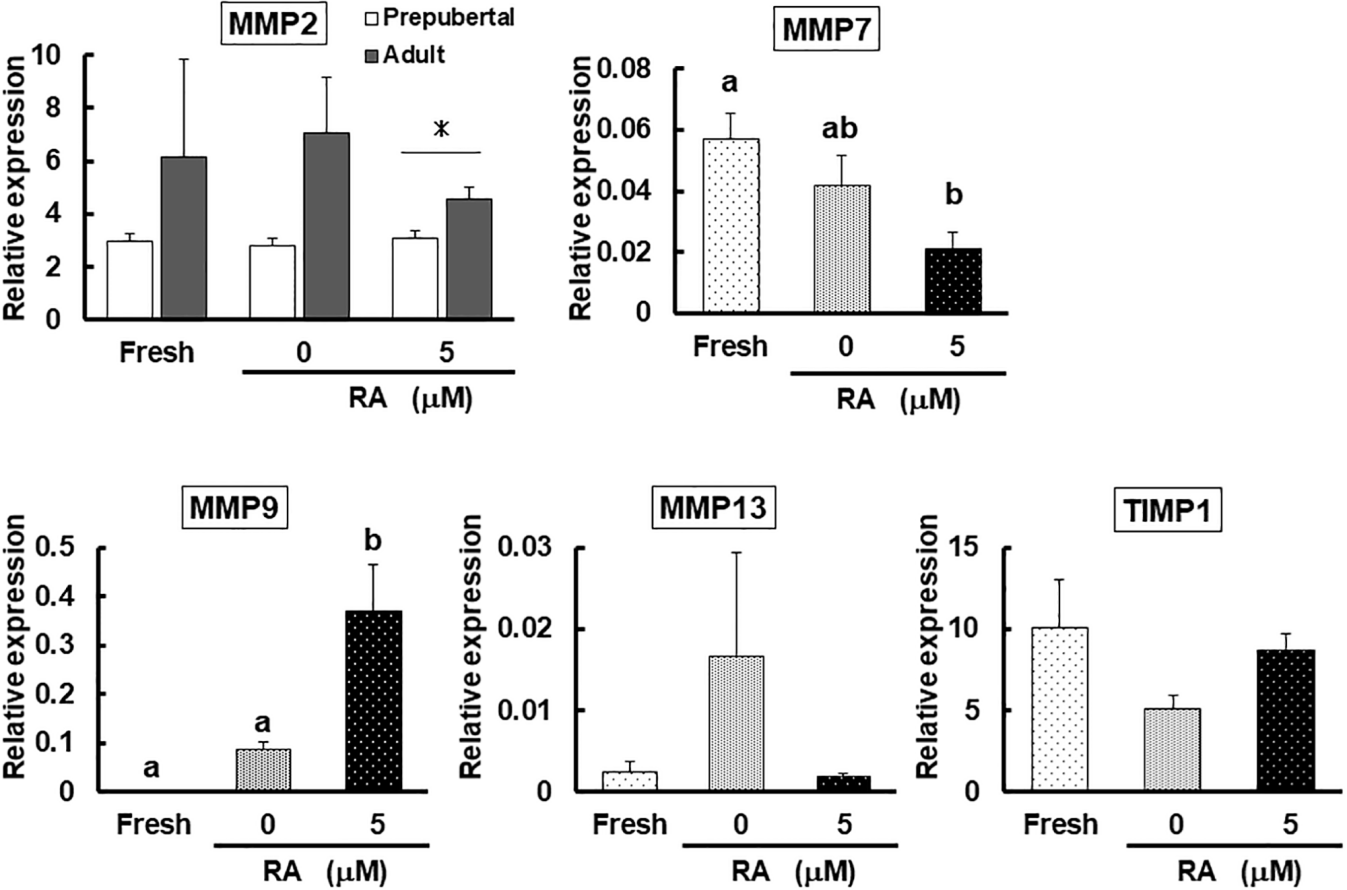
Influence of RA on MMPs and TIMP mRNA expression. Relative mRNA levels of *MMP2*, *MMP7*, *MMP9*, *MMP13 and TIMP1* (normalized to the internal control *GADPH*) in cat ovarian tissue cultured for 0 (fresh) and 7 d with 0 (control) or 5 μM RA. The mRNA level of each gene was shown as mean of 2^-ΔCT^ ± SEM. Different letters indicate differences among treatments (*P* < 0.05). mRNA levels of *MMP2* in prepubertal and adult animals are shown separately, as there was a significant difference (*P* < 0.05) between the two age groups in samples incubated with 5 μM RA (asterisk). With the exception of *MMP2* (n = 4 for each age group), there were eight replications for each gene.

**Fig 4:**
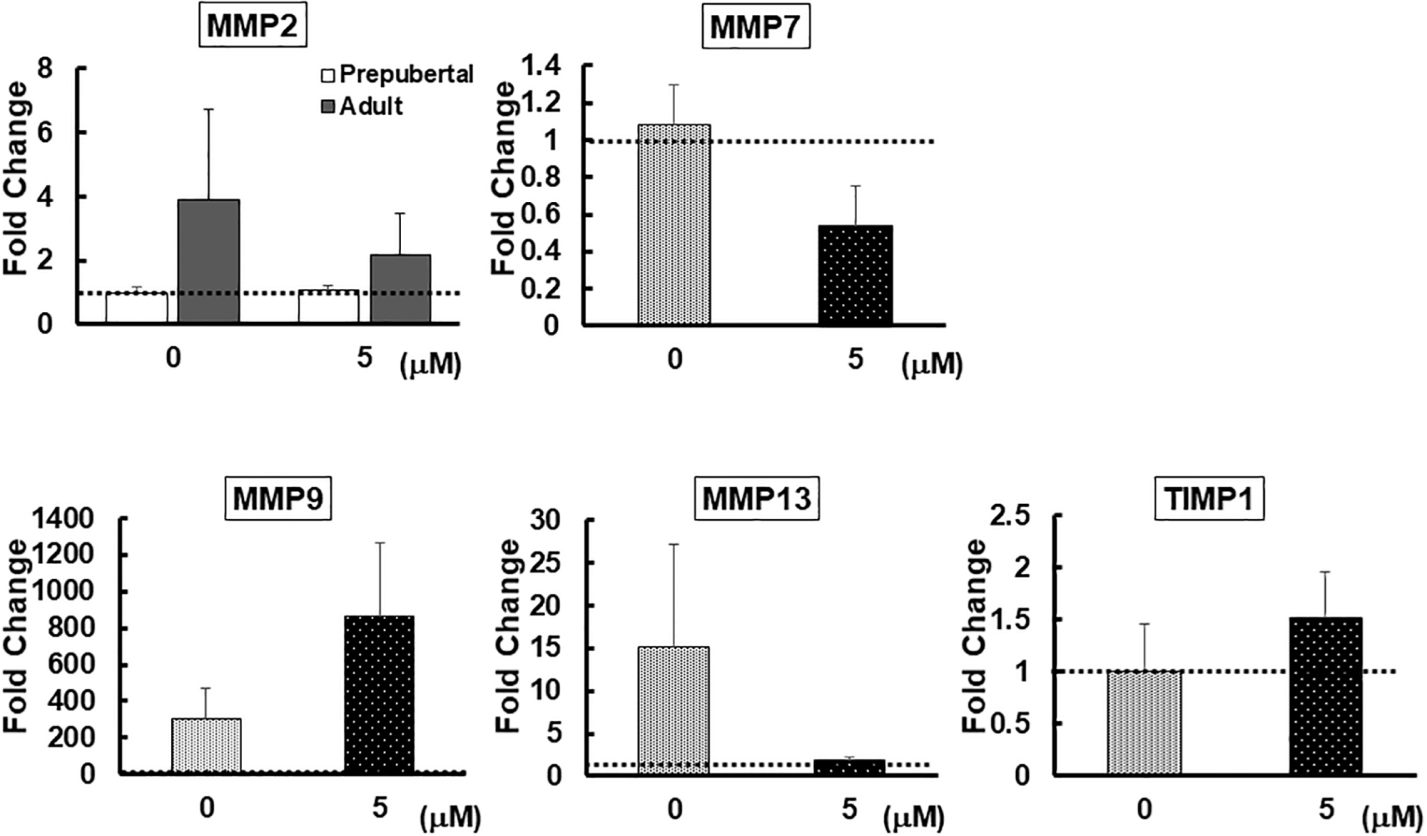
Fold change in *MMP2*, *MMP7*, *MMP9*, *MMP13 and TIMP1* expression relative to fresh tissue. Fold changes of *MMP2* in prepubertal and adult tissue are shown separately. Values were normalized to the values of the fresh tissue, set at 1 as indicated by the dotted line. With the exception of *MMP2* (n = 4 for each age group), there were 8 replications for each gene.

In all cases, *MMP1* and *MMP3* mRNA levels were non-detectable or negligible (data not shown). RA supplementation influenced (*P* < 0.05) mRNA expression of *MMP 7* and *9*, but not *MMP13* or *TIMP1* (Fig 3). The level of *MMP7* transcripts was lower (*P* < 0.05) in cortices incubated with 5 μM RA than that of fresh tissue, but similar to the control (0 μM) (Fig 3). By contrast, 5 μM RA stimulated *MMP9* expression nearly 900-fold compared to the fresh counterpart (*P* < 0.01) and 5-fold more (*P* < 0.01) than the control (Figs 3 and 4). Although the expression level of *MMP2* after RA supplementation was higher in adult than prepubertal animals, there were no differences in mRNA expression of this gene among fresh and culture treatments in both age groups (Fig 3).

### Study 3: Influence of RA on MMP9 protein expression

**Fig 5:**
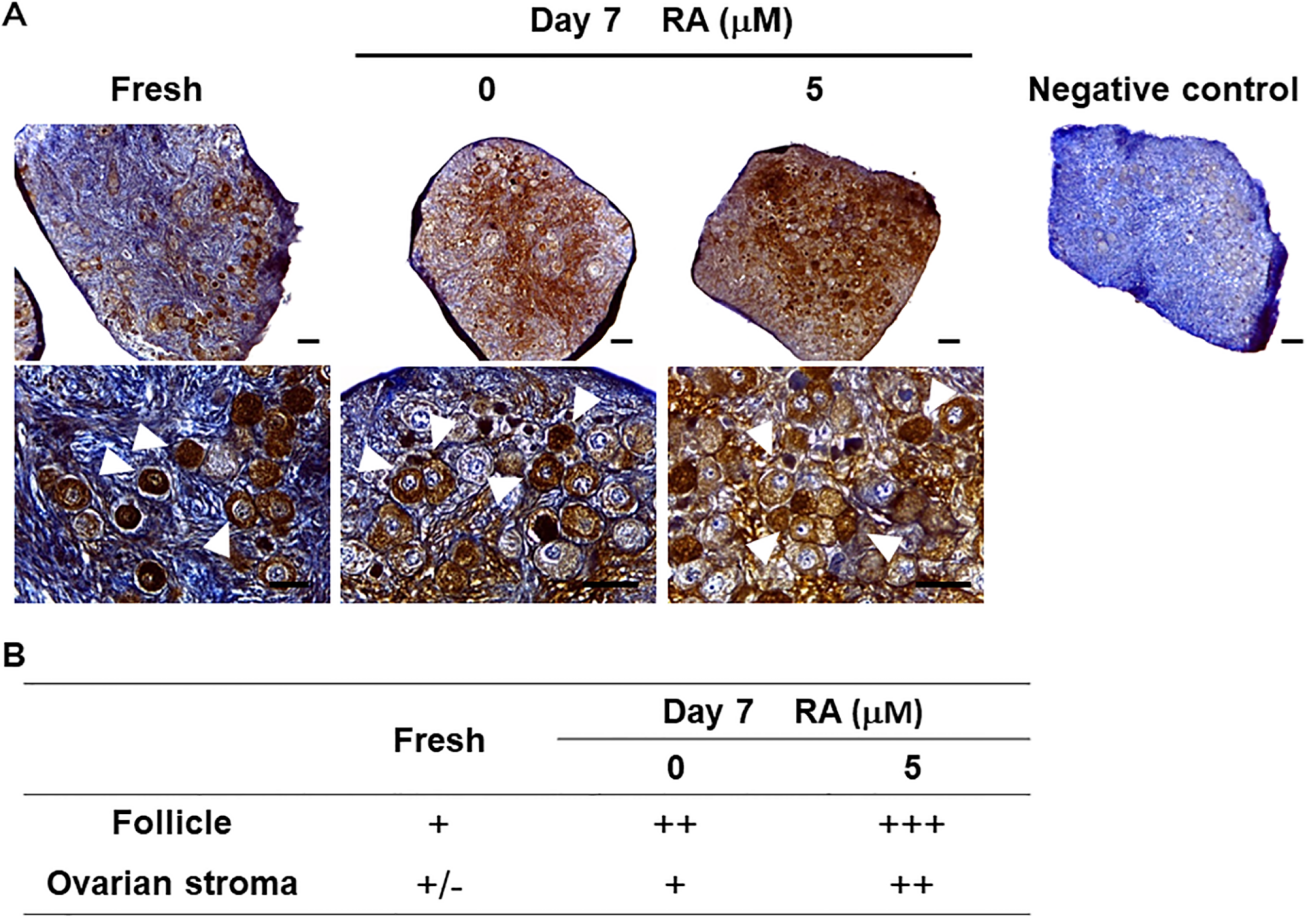
Influence of RA on MMP9 protein expression in cat ovarian tissue. (A) Immunohistographs of MMP9 expression in cat ovarian tissue cultured for 0 (fresh) and 7 d with 0 (control), 1 or 5 μM RA. For the negative control, each primary antibody was replaced with normal rabbit IgG. Bars in upper panels (low magnification) = 100 μm and those in bottom panels (high magnification) = 50 μm. Arrow heads indicate stained follicles. (B) Immunohistochemistry results of MMP9 expression in the cat ovary. Degree of staining was graded subjectively as very strong (+++), strong (++), positive (+) or weak/varied (+/−).

Immunohistochemistry analysis revealed that MMP9 protein expression increased after *in vitro* culture compared to fresh tissue and was clearly more so with RA supplementation (Fig 5). Cytoplasmic localization of MMP9 in fresh tissue was mostly restricted to the oocyte and granulosa cells. However, stromal cells also were immunostained with MMP9 in cultured cortices, and both stromal and granulose cells as well as the oocyte were stained more strongly than in fresh and control tissues (Fig 5). Again, there was no donor age effect, with these protein expression patterns being both common and similar between prepubertal and adult cat ovaries.

## Discussion

During growth, the ovarian follicle undergoes a marked increase in surface area facilitated by remodeling of the follicular wall and adjacent ECM, a process controlled by MMPs and TIMPs [2,3]. The vitamin A metabolite RA has been demonstrated to influence MMP expression in a variety of non-reproductive cell types [6,7] as well as the oocyte and embryo [5,8,9]. Particularly interesting is that RA concentration within human follicular fluid increases with follicle growth, with the amount of RA positively correlated to oocyte competence [9]. In the pig, this phenomenon is demonstrated by RA supplementation improving *in vitro* oocyte maturation and subsequent blastocyst formation [15]. Using a non-rodent model, the domestic cat, we discovered for the first time that RA influenced ovarian follicle development, especially at the primordial stage. Specifically, the presence of 5 μM of RA stimulated growth of primordial follicles enclosed within ovarian cortex in the described culture microenvironment. Furthermore, the potential mechanism of action was through enhanced *MMP9* mRNA and protein expression and down-regulation of *MMP7* mRNA expression. Clearly this specific retinoid plays a key role not only in oocyte and embryo development, but also in early stages of follicular growth.

The mechanism by which MMPs are manifested in reaction to RA presence depends on cell type [6,7,15,16]. For example, RA provokes expression of *MMP2 in vitro* in retinal pigment epithelium cells [15], but down-regulates this same MMP in human arterial smooth muscle cells and corneal keratocytes [16]. Likewise, RA induces neuronal cellular differentiation by up-regulating *MMP9* [6], but by inhibiting *MMP2* and *MMP9* expression in human arterial smooth muscle cells [7]. In the present study, supplementing RA into culture medium enhanced both mRNA and MMP9 protein production. The increase of *MMP9* transcript level in cortices exposed to the highest RA concentration was prodigious (900 fold compared to the fresh control). This effect was validated by the immunohistochemistry analysis that also confirmed stimulated MMP9 protein expression in ovarian cortex treated with RA. These observations also were in alignment with our earlier report that there is a temporal expression of gradually increased *MMP9* transcript during folliculogenesis in this model system [4]. Thus, these collective observations provided confidence that at least one mechanism for how RA promoted follicle growth in the cat was by stimulating both MMP9 mRNA and protein expression.

There was a down-regulation of *MMP7* in cortices incubated with RA, although expression changes were relatively modest (a reduction of about 50% compared to the magnitude of *MMP9* enhancement and the fresh control) and no difference were observed among culture groups. Presence of MMP7 in the ovary has been reported in primates [17,18] and the cat [4], but without specific evidence for the role of this enzyme in the follicle. In our earlier discovery of *MMP7* mRNA in the cat ovary [4], we also found detectable levels at all follicular phases. Transcript levels drastically increased as the follicles transitioned from primordial to primary stage and remained consistent from the primary through antral stages [4]. Thus, reduction of MMP7 in 5 μM RA treatment was unexpected since cortices incubated with vitamin A metabolite contained more primary and secondary stage follicles than fresh and cultured control groups. So far, there has been little information on roles of MMP7 on ovarian follicle development [19]. Former studies have associated MMP7 with ovarian cancer invasion [20]. MMP7 also has been shown to cleave membrane-bound Fas ligand (FasL) into soluble FasL as well as the precursor of tumor necrosis factor (TNF)-alpha precursor to soluble TNF-alpha, both of which result in apoptosis of native cells [19]. Conversely, MMP 7 has been shown to degrade insulin like growth factor (IGF) binding protein, and thus, increasing the bioavailability of IGF and enhancing cancer cell proliferation [19]. Because MMP7 plays roles in both proliferation and apoptotic process, studies to further elucidate the mechanisms by which this proteolytic enzyme influence ovarian follicle development is warrant.

The functions of MMPs and their tissue inhibitors in ECM remodeling has been characterized for advanced secondary and antral follicles in the mouse, rat, cow, and human [2]. However, there is little knowledge available on the role(s) of MMPs in development of primitive (primordial and primary) follicles. It is known that stimulating MMP2 activities and *MMP10* and *MMP12* mRNA expression improves structural integrity of the basement membranes of the isolated mouse primary follicle while sustaining *in vitro* survival [21,22]. It also has been suggested that MMPs assist in cleaving ECM proteins to release ECM-bound growth factors (e.g., fibroblast growth factor [FGF]) [23]. That reaction, in turn, promotes cell growth and differentiation while expanding space for cell proliferation and migration [23]. These observations also suggested two directions for future study, one being examining how RA influences ECM organization, specifically events that allow the follicle to grow and permit normal oocyte development within the ovary. Because it is possible that some growth factors are involved in RA-induced follicle activation, it also would be worthwhile to evaluate the influence of RA in combination with well known growth factors (e.g., FGF) on follicle development success at early growth stages.

It was worth noting that there was no age of donor impact on our evaluation of RA effects. We originally hypothesized that ovaries from prepubertal and adult cats may respond differently because our earlier studies revealed an age influence on ability of stromal cells to proliferate in response to EGF during *in vitro* culture [13]. However, unlike for EGF, the impact of RA on *in vitro* follicle growth and MMPs expression was age-independent. Although treatment of RA showed higher expression of *MMP2* in adult than prepubertal, implicating the higher sensitivity of *MMP2* toward RA in adult ovary, relative expression level of this gene did not differ among treatments in both age groups. This observation was consistent with earlier observations from our laboratory showing no age effect on MMP expression patterns during cat ovarian folliculogenesis [4]. Therefore, based on all investigations to date, it appears that age of donor is not a significant factor in driving how MMPs regulate ovarian follicular development.

In conclusion, findings indicated that RA promoted *in vitro* activation of cat primordial follicles enclosed within the ovarian cortex, via upregulation of MMP9 and down regulation of MMP7. This is a significant contribution to our long-term goal to rescue the maternal genome using a portion of the vast numbers of ovarian premature follicles that never develop or ovulate [4,10,12,13]. The present study specifically provides more information on what regulates early follicular stage development. Such fundamental, comprehensive knowledge can then be used to create a physical microenvironment that efficiently mimics *in vivo* conditions to facilitate follicle development.

## References

[1] Eppig JJ, O’Brien MJ. Development in vitro of mouse oocytes from primordial follicles. Biol Reprod. 1996;54: 197–207. 10.1095/biolreprod54.1.197 8838017

[2] Rodgers RJ, Irving-Rodgers HF, Russell DL. Extracellular matrix of the developing ovarian follicle. Reproduction. 2003;126: 415–24. 10.1530/rep.0.1260415 14525524

[3] Curry TE, Osteen KG. The matrix metalloproteinase system: changes, regulation, and impact throughout the ovarian and uterine reproductive cycle. Endocr Rev. 2003;24: 428–65. 10.1210/er.2002-0005 12920150

[4] Fujihara M, Yamamizu K, Wildt DE, Songsasen N. Expression pattern of matrix metalloproteinases changes during folliculogenesis in the cat ovary. Reprod Domest Anim. 2016;51: 717–25. 10.1111/rda.12736 27484055

[5] Bowles J, Koopman P. Retinoic acid, meiosis and germ cell fate in mammals. Development. 2007;134: 3401–11. 10.1242/dev.001107 17715177

[6] Chambaut-Guérin AM, Hérigault S, Rouet-Benzineb P, Rouher C, Lafuma C. Induction of matrix metalloproteinase MMP-9 (92-kDa gelatinase) by retinoic acid in human neuroblastoma SKNBE cells: relevance to neuronal differentiation. J Neurochem. 2000;74: 508–17. 10.1046/j.1471-4159.2000.740508.x 10646501

[7] Axel DI, Frigge A, Dittmann J, Runge H, Spyridopoulos I, Riessen R, et al All-trans retinoic acid regulates proliferation, migration, differentiation, and extracellular matrix turnover of human arterial smooth muscle cells. Cardiovasc Res. 2001;49: 851–62. 10.1016/S0008-6363(00)00312-6 11230985

[8] Ikeda S, Kitagawa M, Imai H, Yamada M. The roles of vitamin A for cytoplasmic maturation of bovine oocytes. J Reprod Dev. 2005;51: 23–35. 10.1262/jrd.51.23 15750294

[9] Pauli SA, Session DR, Shang W, Easley K, Wieser F, Taylor RN, et al Analysis of follicular fluid retinoids in women undergoing in vitro fertilization: retinoic acid influences embryo quality and is reduced in women with endometriosis. Reprod Sci. 2013;20: 1116–24. 10.1177/1933719113477487 23427183 PMC3745715

[10] Songsasen N, Comizzoli P, Nagashima J, Fujihara M, Wildt D. The domestic dog and cat as models for understanding the regulation of ovarian follicle development in vitro. Reprod Domest Anim. 2012;47: 13–18. 10.1111/rda.12067 23279457 PMC3579211

[11] Wildt DE, Swanson W, Brown J, Sliwa A, Vargas A. Biology and Conservation of Wild felids M DW, L AJ, editors. Felids ex situ: managed programmes, research and species recovery. New York: Oxford University Press; 2010.

[12] Fujihara M, Comizzoli P, Wildt DE, Songsasen N. Cat and dog primordial follicles enclosed in ovarian cortex sustain viability after in vitro culture on agarose gel in a protein-free medium. Reprod Domest Anim. 2012;47 Suppl 6: 102–8. 10.1111/rda.12022 23279476 PMC3965327

[13] Fujihara M, Comizzoli P, Keefer CL, Wildt DE, Songsasen N. Epidermal growth factor (EGF) sustains in vitro primordial follicle viability by enhancing stromal cell proliferation via MAPK and PI3K pathways in the prepubertal, but not adult, cat ovary. Biol Reprod. 2014;90: 86 10.1095/biolreprod.113.115089 24554736

[14] Schmittgen TD, Livak KJ. Analyzing real-time PCR data by the comparative C(T) method. Nat Protoc. 2008;3: 1101–8. 10.1038/nprot.2008.73 18546601

[15] Gao Z, Huo L, Cui D, Yang X, Zeng J. The expression of bone morphogenetic protein 2 and matrix metalloproteinase 2 through retinoic acid receptor beta induced by all-trans retinoic acid in cultured ARPE-19 cells. Lewin AS, editor. PLoS One. 2016;11: e0150831 10.1371/journal.pone.0150831 26967733 PMC4788292

[16] Abidin FZ, Gouveia RM, Connon CJ. Application of retinoic acid improves form and function of tissue engineered corneal construct. Organogenesis. 2015;11: 122–36. 10.1080/15476278.2015.1093267 26496651 PMC4879898

[17] Chaffin CL, Stouffer RL. Expression of matrix metalloproteinases and their tissue inhibitor messenger ribonucleic acids in macaque periovulatory granulosa cells: time course and steroid regulation. Biol Reprod. 1999;61: 14–21. 10.1095/biolreprod61.1.14 10377026

[18] Meinel S, Blohberger J, Berg D, Berg U, Dissen GA, Ojeda SR, et al Pro-nerve growth factor in the ovary and human granulosa cells. Horm Mol Biol Clin Investig. 2015;24: 91–9. 10.1515/hmbci-2015-0028 26457789 PMC4760111

[19] Ii M, Yamamoto H, Adachi Y, Maruyama Y, Shinomura Y. Role of Matrix Metalloproteinase-7 (Matrilysin) in Human Cancer Invasion, Apoptosis, Growth, and Angiogenesis. Exp Biol Med. SAGE PublicationsSage UK: London, England; 2006;231: 20–27. 10.1177/153537020623100103 16380641

[20] Al-Alem L, Curry TE Jr. Ovarian cancer: involvement of the matrix metalloproteinases. Reproduction. NIH Public Access; 2015;150: R55–64. 10.1530/REP-14-0546 25918438 PMC4955511

[21] Murray AA, Molinek MD, Baker SJ, Kojima FN, Smith MF, Hillier SG, et al Role of ascorbic acid in promoting follicle integrity and survival in intact mouse ovarian follicles in vitro. Reproduction. 2001;121: 89–96. 10.1530/rep.0.1210089 11226031

[22] Tagler D, Makanji Y, Tu T, Bernabé BP, Lee R, Zhu J, et al Promoting extracellular matrix remodeling via ascorbic acid enhances the survival of primary ovarian follicles encapsulated in alginate hydrogels. Biotechnol Bioeng. 2014;111: 1417–29. 10.1002/bit.25181 24375265 PMC4232184

[23] Page-McCaw A, Ewald AJ, Werb Z. Matrix metalloproteinases and the regulation of tissue remodelling. Nat Rev Mol Cell Biol. 2007;8: 221–33. 10.1038/nrm2125 17318226 PMC2760082

